# Multi-Year Comparison of Community- and Species-Level West Nile Virus Antibody Prevalence in Birds from Atlanta, Georgia and Chicago, Illinois, 2005–2016

**DOI:** 10.4269/ajtmh.21-1086

**Published:** 2022-12-26

**Authors:** Joseph R. McMillan, Gabriel L. Hamer, Rebecca S. Levine, Daniel G. Mead, Lance A. Waller, Tony L. Goldberg, Edward D. Walker, Jeffrey D. Brawn, Marilyn O. Ruiz, Uriel Kitron, Gonzalo Vazquez-Prokopec

**Affiliations:** ^1^ Program in Population Biology, Ecology and Evolution, Emory University, Atlanta, Georgia;; ^2^ Department of Entomology, Texas A&M University, College Station, Texas;; ^3^ Southeastern Cooperative Wildlife Disease Study, University of Georgia, Athens, Georgia;; ^4^ Department of Biostatistics and Bioinformatics, Rollins School of Public Health, Emory University, Atlanta, Georgia;; ^5^ Department of Pathobiological Sciences, School of Veterinary Medicine, University of Wisconsin–Madison, Madison, Wisconsin;; ^6^ Department of Microbiology and Molecular Genetics, Michigan State University, East Lansing, Michigan;; ^7^ Department of Natural Resources and Environmental Sciences, University of Illinois Champaign–Urbana, Urbana, Illinois;; ^8^ Department of Pathobiology, University of Illinois Champaign–Urbana, Urbana, Illinois;; ^9^ Department of Environmental Sciences, Emory University, Atlanta, Georgia

## Abstract

West Nile virus (WNV) is prevalent in the United States but shows considerable variation in transmission intensity. The purpose of this study was to compare patterns of WNV seroprevalence in avian communities sampled in Atlanta, Georgia and Chicago, Illinois during a 12-year period (Atlanta 2010–2016; Chicago 2005–2012) to reveal regional patterns of zoonotic activity of WNV. WNV antibodies were measured in wild bird sera using ELISA and serum neutralization methods, and seroprevalence among species, year, and location of sampling within each city were compared using binomial-distributed generalized linear mixed-effects models. Seroprevalence was highest in year-round and summer-resident species compared with migrants regardless of region; species explained more variance in seroprevalence within each city. Northern cardinals were the species most likely to test positive for WNV in each city, whereas all other species, on average, tested positive for WNV in proportion to their sample size. Despite similar patterns of seroprevalence among species, overall seroprevalence was higher in Atlanta (13.7%) than in Chicago (5%). Location and year of sampling had minor effects, with location explaining more variation in Atlanta and year explaining more variation in Chicago. Our findings highlight the nature and magnitude of regional differences in WNV urban ecology.

## BACKGROUND

West Nile virus (WNV) is a globally distributed arbovirus transmitted among birds by *Culex* spp. mosquitoes. WNV is the most prevalent zoonotic arbovirus in the continental US, although enzootic transmission intensity throughout the country varies over space and time.^1^^,^^2^ Evidence from field studies suggests that variability in vector–host contacts is a biologically significant regional driver of WNV transmission among reservoir hosts (i.e., birds),^3^^,^^4^ the primary urban vectors of WNV,^5^ and spillover into human populations.^6^^,^^7^ Overall, high infection rates in mosquitoes are generally predictive of WNV clinical cases in humans.^8^ However, WNV incidence in humans (including neuroinvasive manifestations) occurs more frequently in the Midwest, Western, and Northeastern US, compared with states in the Southeast and Northwest,^9^ despite documented and intense enzootic WNV activity in all regions of the US. To address variability of enzootic and epidemic WNV transmission in the US, the growing availability of longitudinal WNV surveillance datasets in the US provides an opportunity to compare WNV infection metrics between regions. Such comparisons may be useful for characterizing drivers of WNV transmission heterogeneities across the US and informing risk of spillover into human populations.

The composition of host reservoir species communities is of particular importance when comparing WNV regional transmission dynamics. Species such as the American robin (*Turdus migratorius*) and house sparrow (*Passer domesticus*) are considered important amplifying hosts of WNV in urban environments in the Midwestern and Eastern US because both are common and competent for WNV,^10^^,^^11^ and *Culex pipiens* complex mosquitos blood feed on these species in excess of their field abundance.^4^^,^^6^^,^^7^^,^^12^ In the Southeastern US, however, an overutilization of relatively incompetent WNV avian species such as northern cardinals (*Cardinalis cardinalis*) and northern mockingbirds (*Mimus polyglottos*) by *Culex quinquefasciatus* has been proposed as an explanation for dampened WNV transmission.^4^ These studies identify epidemiologically important avian WNV amplification species based on blood meal analysis to determine high utilization of WNV-competent species.^7^^,^^13^ Identifying interannual and regional variability of transmission by comparing infection prevalence estimates within avian communities has received less attention. The lack of information on WNV infection data in avian communities is partly due to the difficulty of appropriate field sampling.^14^ The period during which infectious WNV viremia is detected in avian blood is typically < 1 week,^4^^,^^15^ and it is difficult to capture and sample wild birds that may be infectious within this timeframe. Instead, birds are commonly tested for the presence and quantity of WNV neutralizing (or blocking) antibodies in serum samples. Detectable antibodies provide an estimate of prior WNV infection, and antibody prevalence (i.e., seroprevalence) has been used to indirectly quantify WNV transmission patterns in host communities.^4^^,^^15^^–^^17^ For example, hatch year birds are susceptible to WNV when maternally acquired antibodies decay within a few days to weeks after hatching,^18^^,^^19^ so that, depending on when a hatch year individual is sampled, the detection of WNV antibodies can provide a serological indicator of a recent exposure event.

Our objective was to analyze retrospective data from two longitudinal US studies of WNV seroprevalence in bird species sampled in Atlanta, Georgia and Chicago, Illinois to assess variability in WNV transmission between the two regions. WNV invaded each city at similar times (∼2000–2002),^20^ and similar viral variants are found in each city;^21^ in general, the enzootic WNV transmission season is longer in Atlanta than Chicago.^20^ Short-term field studies in each city have identified active and intense WNV enzootic cycles as well as important ecological and climatic drivers of transmission. In Chicago, avian-specific studies documented variability of WNV seroprevalence in sampled bird communities,^15^ the absence of a dilution or amplification effect between avian species diversity and WNV infection rates in mosquitoes,^22^ the influence of roosting behaviors on the bite force of local mosquitoes,^23^ and the importance of American robins and house sparrows as amplifying hosts of WNV.^6^ In Atlanta, similar field study designs and laboratory methodologies as used in Chicago revealed blood meal host shifts from American robins to northern cardinals as a possible transmission suppression mechanism,^4^^,^^14^ an amplification effect between avian species diversity and WNV infection rates in mosquitoes,^3^ no discernable pattern of blood feeding preferences for certain species in controlled blood feeding trials,^24^ and defined the role of susceptible host availability on vectorial capacity of locally abundant *Culex *spp. mosquitoes.^25^

We made no a priori assumptions regarding overall seroprevalence estimates between each city; however, we broadly hypothesized that species-specific patterns of WNV seroprevalence would vary between cities. Of particular interest was the comparison of WNV seroprevalence estimates in American robins and northern cardinals since these species have been identified as important hosts in each city.^4^^,^^6^^,^^26^

## METHODS

### Avian WNV surveillance methodology.

From 2005 to 2012 and from 2010 to 2016, avian communities in Chicago and Atlanta, respectively, were sampled for evidence of WNV infection in birds in numerous urban habitats.^4^^,^^26^^,^^27^ Sampling generally took place weekly from May to October (with some variation across years and cities) to monitor serostatus. Birds were captured using ground-level mist nets. Individuals were identified to sex, age, and species (when possible) following Pyle,^28^ banded, and released. Age, specifically of hatch year birds, was identified using numerous characteristics such as, but not limited to, plumage, bill, and gape characteristics. Sex designations in hatch year birds were seldom made due to the limits of using plumage as an indicator of sex at young ages. Up to 200 μL of blood was collected via jugular venipuncture from birds that weighed > 10 g and were in suitable physical condition (e.g., no visible injuries or signs of stress).

All sera were tested for IgY (an avian immunoglobulin functionally similar to the mammalian IgG). Specifically, all sera from Chicago (2005–2012) and from Atlanta (2010–2013) were tested using blocking ELISA (b-ELISA) techniques following Hamer et al.,^15^ whereas sera from Atlanta (2014–2016) were tested using serum neutralization (SNT) techniques^29^ due to logistical constraints. Using ELISA methods, percent inhibitions ≥ 60% were considered WNV positive, whereas for SNT methods, inhibition of cytopathic effects in cell culture at titers ≥ 1:8 were considered WNV positive. The WNV b-ELISA was designed similarly to a previously developed ELISA for St. Louis encephalitis virus, which showed good agreement between b-ELISA and a plaque reduction neutralization test.^30^ The degrees to which sera test positive for WNV (i.e., the endpoint titer of the test) under each method are not directly comparable unless performed side by side. However, in pilot analyses comparing WNV seroprevalence in a subsample of Atlanta’s data, we detected no difference in WNV serostatus between known ELISA results and the tested SNT method. Therefore, we limited our analyses to the specific serostatus of individuals, and we did not investigate differences in reported antibody titers among individuals within or between seasons.

All work was approved by the following: Chicago (2005–2012): University of Illinois animal use protocol no. 03034 and Institutional Animal Care and Use Committee at Michigan State University, animal use form no. 12/03-152-00; Atlanta (2010–2016): U.S. Geological Society permit no. 23673, Georgia Department of Natural Resources scientific collection permit no. 23772, Emory Institutional Animal Care and Use Committee approval DAR-2003079.

### Data analysis.

Two-sample tests of proportions were used to compare WNV seroprevalence rates between each city without implementing any data restrictions or accounting for variability between years, locations, and species. Further comparisons of WNV seroprevalence within and between cities used a combination of generalized linear mixed-effects models (GLMMs).

The serostatus of a sampled individual, modeled as 0 (seronegative) or 1 (seropositive), was chosen as the primary unit of analysis. All generalized linear models and GLMMs contained an intercept offset to account for variability of sampling effort within and between cities (Supplemental Figure 1, effort calculated as the natural log transformation of number of sampling events per month each year). We first compared variation in WNV seroprevalence by age, time (i.e., week, month, year), and location of sampling (i.e., site name) within cities using single-term logistic regressions; these explorations were included to determine how variable form affected model performance (e.g., time of sampling recorded as fixed, categorical versus continuous, or integer variables), with forms showing the greatest reduction in the Aikaike information criteria (ΔAIC) selected for consideration within multivariate models. We then used multivariate binomial GLMMs to compare WNV seroprevalence between years and species in each city independently and between cities when the datasets overlapped (2010–2012). For GLMMs, we limited our analyses to species sampled in each city with at least one data point across all years and sites; we additionally restricted our analyses to samples collected during WNV detection periods in mosquitoes from each city (approximately June to October). To reduce pseudoreplication due to recaptures of individuals, we included only the first sample from a captured individual in each analysis. Additionally, we excluded data for individuals that could not be identified to age or species or for which identifying information was ambiguous. Following prior findings that found no statistically significant relationship between sex and WNV serostatus,^4^ we did not include sex in any model.

After univariate analyses, we chose to model time as the unit of month rather than week because data aggregated to the week level was too sparse for adequate model performance (i.e., there were many weeks in which few or no birds were captured). We also chose to model species as a crossed random effect,^31^ which allowed us to compare WNV seroprevalence across all sampled species rather than to a reference species if species were modeled as a fixed effect.^32^ The remaining random effect terms, year and sampling location (henceforth termed location), were modeled as nested effects because not all sites were sampled in all years in each city. The same model formulation and structure were held constant for each city’s analysis. In the Atlanta dataset, location had previously been investigated as a fixed effect on a microhabitat scale. Because preliminary analyses revealed only minor variation in WNV seroprevalence in sites within the same urban park space, data from within-park microhabitats were aggregated to a larger “park” location variable. The same level of microhabitat subsampling did not need be addressed in the Chicago dataset.

We implemented all GLMMs in R v. 3.6^33^ using the glmmTMB package.^34^ We generated WNV seroprevalence predictions and assessed random effects terms from each GLMM using a combination of functions available in the ggeffects and sjPlot packages.^35^^,^^36^

## RESULTS

### Summary data and statistics.

**Figure 1.**
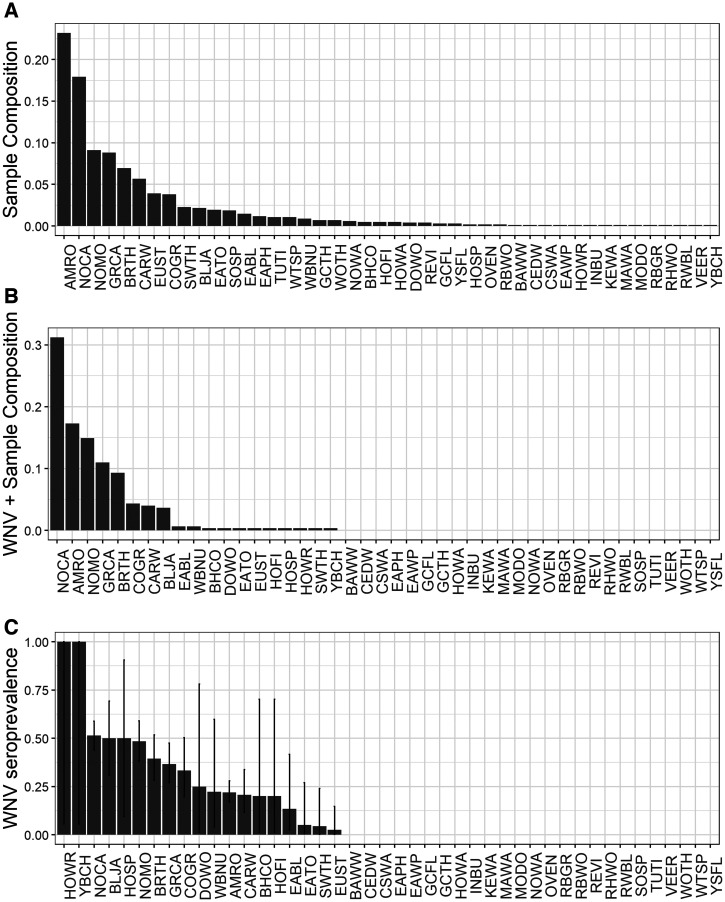
Total (**A**) and WNV–positive (**B**) sample composition of birds captured and sampled for WNV antibodies in Atlanta, Georgia, 2010–2016. (**C**) Estimated WNV seroprevalence (±SE) for each species sampled in Atlanta.

**Figure 2.**
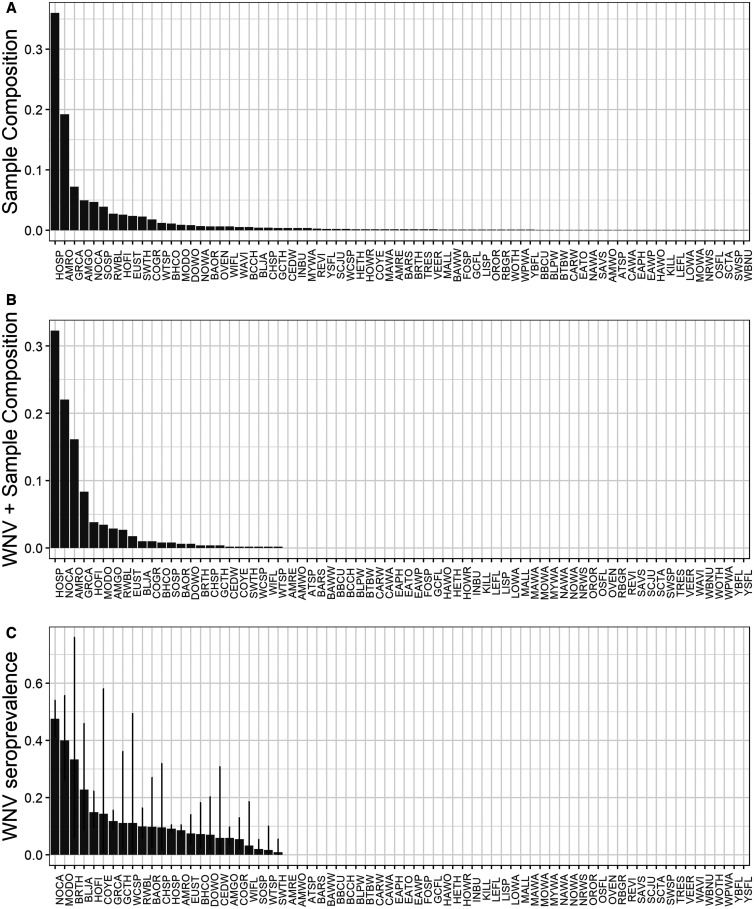
Total (**A**) and WNV–positive (**B**) sample composition of birds captured and sampled for WNV antibodies in Chicago, Illinois, 2005–2012. (**C**) Estimated WNV seroprevalence (±SE) for each species sampled in Chicago.

**Figure 3.**
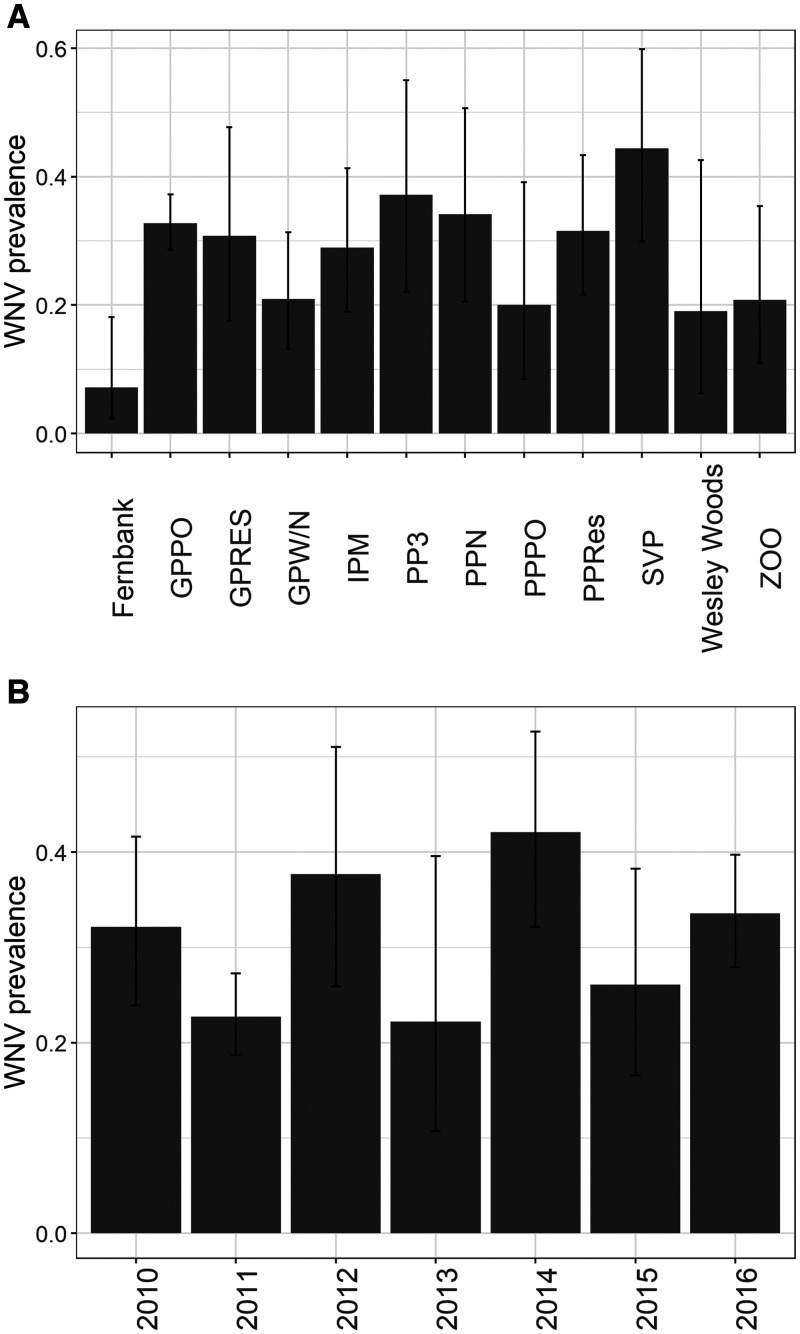
(**A**) Estimated WNV seroprevalence in birds by sampling location in Atlanta, Georgia, 2010–2016. (**B**) Estimated WNV seroprevalence in birds by year in Atlanta, Georgia, 2010–2016. Bars represent the estimate; lines represent the 95% CI of the estimate. Sampling location maps and descriptions can be found in the referenced Atlanta-specific studies (see* Background*).

**Figure 4.**
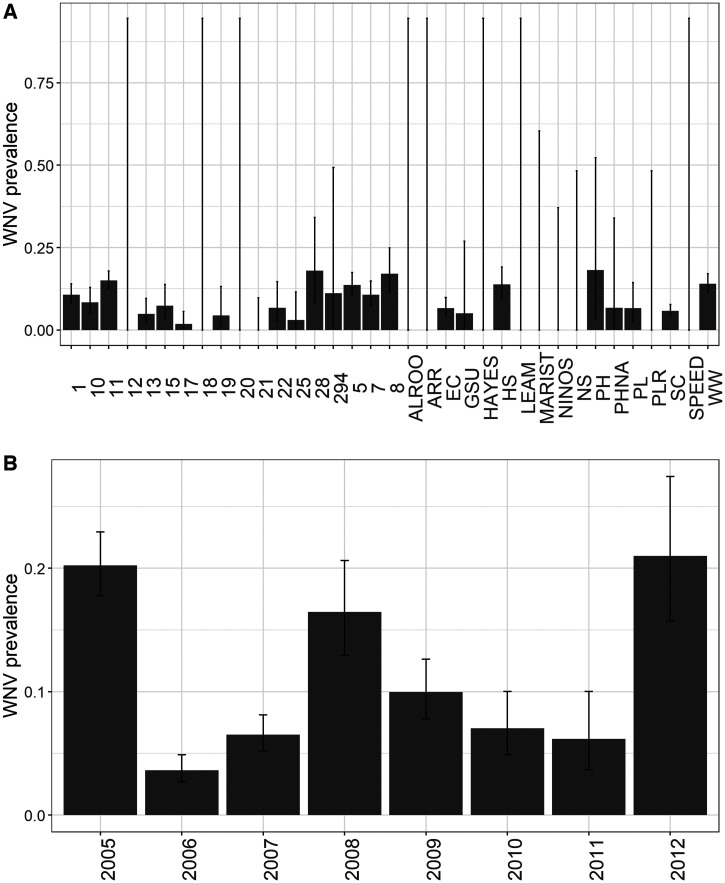
(**A**) Estimated WNV seroprevalence in birds by location in Chicago, Illinois, 2005–2012. (**B**) Estimated WNV seroprevalence in birds by year in Chicago, Illinois, 2005–2012. Bars represent the estimate; lines represent the 95% CI of the estimate. Sampling location maps and descriptions can be found in the referenced Chicago-specific studies (see* Background*).

The combined dataset contained 6,254 blood samples from 86 bird species in 47 unique study sites across 12 collection years (Atlanta: 44 spp., 12 locations, 7 years, *N* = 1,022; Chicago: 77 spp., 35 locations, 8 years, *N* = 5,232). In each city, evidence of prior WNV exposure was widely prevalent across species (Figures 1 and 2, Atlanta: 43% of all species; Chicago: 29% of all species), across locations within each city (Figures 3 and 4, Atlanta: 100% of locations; Chicago: 63% of locations), and across years (Figures 3 and 4, 100% of years in each city). A summary of all species sampled in each city is shown in Supplemental Table 1.

The composition of avian communities sampled was similar between cities, with most samples coming from year-round resident species followed by summer breeding (Table 1, resident status classified following ebird.org). More summer breeding species were sampled in Chicago than in Atlanta; however, in each city most WNV positive samples and species were identified as year-round residents. In general, resident species were sampled frequently across years and were widely distributed across all sampled sites in each city. The resident species American robins, house sparrows, American goldfinches (*Spinus tristis*), and northern cardinals and the summer breeding species gray catbird (*Dumetella carolinensis*) were the most frequently sampled species in Chicago. The resident species American robins, northern mockingbird, northern cardinal, and brown thrasher (*Toxostoma rufum*) and the summer breeding species gray catbird were the most frequently sampled species in Atlanta.

**Table 1:** Summary results of total sampling and WNV seroprevalence among species classified as year-round residents, summer breeders, migrants, and winter residents in Atlanta, Georgia and Chicago, Illinois

Migratory status	Atlanta, Georgia, 2010–2016				Chicago, Illinois, 2005–2012			
	Sample proportion (*N* = 1,022)	Tested species (*N* = 46)	WNV-positive samples (*N* = 301)	WNV-positive species (*N* = 19)	Sample proportion (*N* = 5,232)	Tested species (*N* = 72)	WNV-positive samples (*N* = 527)	WNV-positive species (*N* = 24)
All year resident	83.5%	54.5%	88.4%	84.2%	82.3%	27.8%	89.0%	58.3%
Summer breeding	11.4%	25.0%	11.3%	10.5%	11.8%	43.1%	10.1%	25.0%
Migratory stopover	3.9%	15.9%	3.3%	5.26%	4.2%	22.2%	0.6%	8.33%
Winter resident	1.2%	4.5%	0.0%	0.0%	1.5%	6.9%	0.4%	8.33%

Overall, WNV seroprevalence was higher in Atlanta than in Chicago (two-sample proportions test, *χ*^2^ = 277.9,* P *< 2.2e−16; Figures 1–4). This difference between cities was consistent for adults (*χ*^2^ = 191.2,* P *< 2.2e−16) and hatch year birds (*χ*^2^ = 41.9,* P *< 9.8e−11) but not for the number of species positive for WNV (*χ*^2^ = 1.3,* P *= 0.26). Higher WNV seroprevalence in Atlanta compared with Chicago remained significant whether restricting data to locations sampled in all years for each city (*χ*^2^ = 136.3,* P *< 2.2e−16), to species shared between cities (see following section for species; *χ*^2^ = 107.0,* P *< 2.2e−16), and to years shared between cities (2010–2012; *χ*^2^ = 66.2,* P *< 4.1e−16). WNV seroprevalence also remained significantly higher in Atlanta than in Chicago when comparing prevalence by the residence status of the avian species (resident: *χ*^2^ = 238.31,* P *< 2.2e−16; summer breeding: *χ*^2^ = 37.58,* P *< 9.1e−10).

At the level of individual species sampled in each city, WNV seroprevalence was higher in Atlanta than in Chicago for three species: American robin (*χ*^2^ = 34.2,* P *< 6e−9), common grackle (*χ*^2^ = 15.7,* P *< 8e−9), and gray catbird (*χ*^2^ = 30.9,* P *< 3e−8). There were no seroprevalence differences between cities for the brown-headed cowbird (*Molothrus ater*), blue jay (*Cyanocitta cristata*), brown thrasher, European starling (*Sturnus vulgaris*), gray-cheeked thrush (*Catharus minimus*), house finch (*Haemorhous mexicanus*), northern cardinal, song sparrow (*Melospiza melodia*), Swainson’s thrush (*Catharus ustulatus*), or white-throated sparrow (*Zonotrichia albicollis*). For all other species, there were not enough total samples (fewer than five) to justify a proportion’s test. When examining trends in sampling frequency and WNV seroprevalence in birds sampled during the overlap period of each dataset, seroprevalence trends are similar.

### Patterns of avian WNV antibody prevalence within each city.

After implementing our data restrictions regarding frequency of sampling within and between each city, six species provide adequate sample sizes to compare across years and habitats: American robin (AMRO), common grackle (COGR), European starling (EUST), gray catbird (GRCA), northern cardinal (NOCA), and Swainson’s thrush (SWTH) Supplemental Table 1). Because SWTH is a migratory species that does not breed in either city, it was not included in any final model. The remaining five species represent resident breeding species and reflect birds exposed during the summer WNV amplification season.

**Figure 5.**
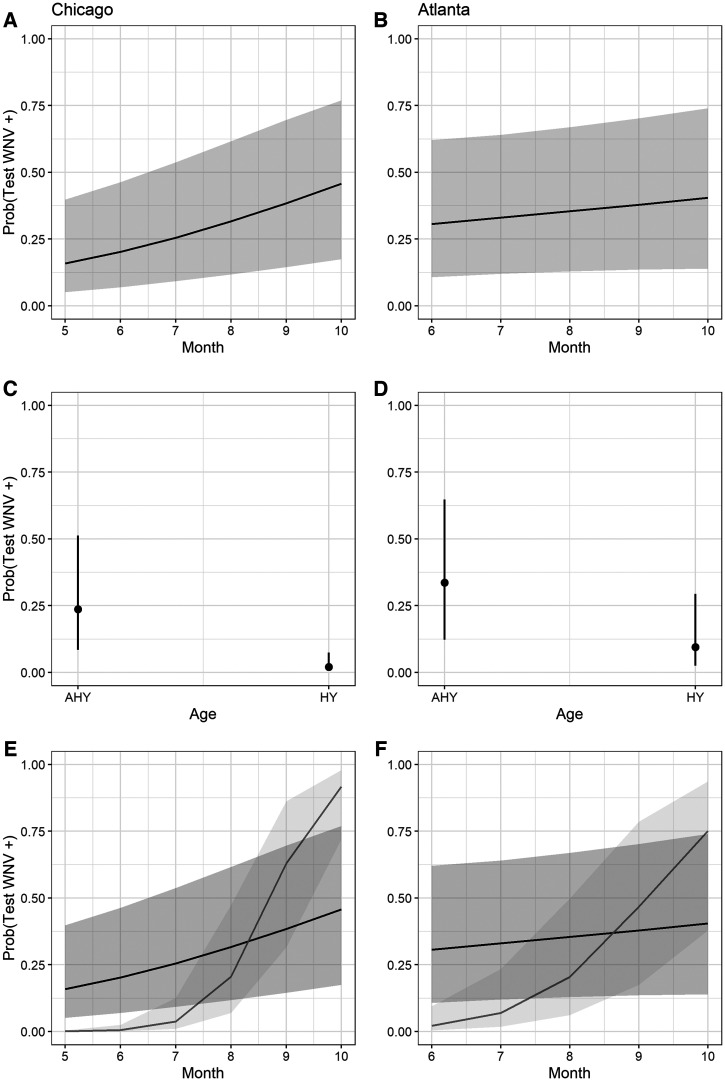
Fixed effect predictions of a single resident bird testing positive for WNV antibodies in Chicago, Illinois, 2005–2012 (**A**, **C**, and **E**) and Atlanta, Georgia, 2010–2016 (**B**, **D**, and **F**). (**A** and **B**) Predicted effect of month on a single bird of any age in any year testing positive for WNV. (**C** and **D**) Predicted effect of age on a single bird of any species in any year testing positive for WNV. (**E** and **F**) The predicted interaction between month and age for a single resident bird in any year testing positive for WNV (black line indicates adults; gray line indicates hatch year birds). Predictions were generated from a binomial generalized linear mixed effects model with WNV antibody positive as the response term, total monthly trapping effort was included as an intercept offset, month, age, and a month-by-age interaction were fixed effect terms, species was a crossed random effect, and year and location were nested random effect terms. Solid lines represent the average prediction, and shaded regions (or bars) indicate the 95% CI of the prediction. AHY indicates hatch year bird; HY indicates hatch year bird.

Using only samples obtained from these five species, each city-specific GLMM demonstrated similarities in patterns of WNV seroprevalence. Consistent with prior North American studies of WNV avian serology, adults were more likely than hatch year birds to test positive for WNV (Table 2, Figure 5), and the likelihood of hatch year birds testing positive for WNV increased throughout a season (Figure 5). However, the probability of detecting WNV antibodies across age, species, year, and location was generally higher in Atlanta than in Chicago during early summer months (Figures 1–5), and the predicted effect of month on the probability of detecting WNV antibodies was only significant in the Chicago GLMM (Table 2). Also, WNV seroprevalence in adult birds remained relatively flat (∼30%) across years, species, and locations in Atlanta, whereas in Chicago, the probability of detecting WNV rose for both age groups across years, species, and locations: from ∼12% in June to 40% in October for adults, and from ∼0% in June to 85% in October for hatch year birds (Figure 5).

**Table 2:** Results from two independent binomial generalized linear mixed-effects models of WNV antibody detection probabilities in birds sampled in Atlanta, Georgia (2010–2016) and Chicago, Illinois (2005–2012)

	Chicago, Illinois, 2005–2012				Atlanta, Georgia, 2010–2016			
Variable	Estimate (95% CI)	SE	*z* Value	Pr(>|*z*|)	Estimate (95% CI)	SE	*z* Value	Pr(>|*z*|)
Intercept	−5.76 (−7.52, −3.99)	0.90	−6.38	1.7e−10	−3.36 (−5.33, −1.40)	1.00	−3.35	0.0008
Month	0.30 (0.11, 0.49)	0.10	3.03	0.002	0.11 (−0.10, 0.32)	0.11	0.98	0.32
Age	−13.2 (−16.4, −10.0)	1.65	−8.03	9.9e−16	−9.75 (−13.4, −6.11)	1.86	−5.24	1.6e−7
Month by age	1.58 (0.49, 1.92)	0.21	7.4	1.4e−13	1.12 (0.66, 1.58)	0.23	4.79	1.7e−6
Random effects	Name	Variance	SD		Random effects	Name	Variance	SD
Location: year	Intercept	0.26	0.51		Year: location	Intercept	0.74	0.86
Year	Intercept	1.35	1.16		Location	Intercept	0.73	0.86
Species	Intercept	0.94	0.97		Species	Intercept	0.84	0.92

In each generalized linear mixed-effects model, WNV serostatus was the response term, total monthly trapping effort was included as an intercept offset, month, age, and a month-by-age interaction were fixed effect terms, species was a crossed random effect term, and year and location were nested random effect terms. Pr(>|z|) = *P* value.

Further comparisons between patterns of WNV seroprevalence in Atlanta and Chicago were assessed using each GLMM’s random effects. Each city-specific GLMM predicted significant heterogeneity in WNV seroprevalence between species; heterogeneity across sampling locations and species was similar in Atlanta whereas variance of species was greatest in Chicago (Table 2). Among the five modeled species in each city, the predicted probability of detecting WNV was highest for NOCA (Supplemental Figures 2 and 3). In Chicago, only NOCA was a significant source of among species variance in WNV antibody prevalence (Supplemental Figure 3, with significance defined by the 95% CI of the random effect estimate, shown as an odds ratio, excluding unity).

The primary difference between the two cities’ GLMM random effects was that location explained more variance in the Atlanta dataset whereas year explained more variance in the Chicago dataset (Table 2, Supplemental Figures 2 and 3). In Chicago, birds sampled in 2006 were the least likely to test positive for WNV, whereas birds sampled in 2012 were the most likely to test positive for WNV. Additionally, differences in WNV detections between 2006 and 2012 were significantly different (the 95% CIs do not overlap) (Supplemental Figure 3).

### Patterns of WNV antibody prevalence between cities: 2010–2012.

Patterns of WNV seroprevalence between Atlanta and Chicago were comparable to the city-specific GLMMs. Preliminary univariate analyses revealed that age, month, an age by month interaction, year (reference: 2010), and city (reference: Atlanta) were all significant predictors of detecting WNV antibodies across species, years, and locations. In particular, the likelihood of detecting WNV antibodies was lower in Chicago than Atlanta (fixed effect; city: Chicago, estimate −0.92, SE 0.20, *z* value 4.68, *P* value 2.9e−6) in the univariate analyses. However, multivariate GLMMs revealed that city itself was neither a significant (*P* > 0.05) nor an informative fixed-effect term (ΔAIC < 2); preliminary analyses also revealed that interactions between city and all other variables overfit the data, often resulting in convergence failures and increased AIC scores. Therefore, the final model comparing WNV antibody prevalence between Atlanta and Chicago included year (as a categorical variable), age, month, and an age-by-month interaction fixed-effect terms and location nested within species as a random effect term (Table 3). Although city was not explicitly modeled, heterogeneity in outcomes between the two cities was assessed using the random effect terms. The inclusion of year as a fixed effect is the main difference in model construction between the overlap and the city-specific GLMMs; this is due to issues surrounding prediction accuracy when there are fewer than five levels for a random effect term.^31^

**Table 3:** Results from a binomial generalized linear mixed-effects models of WNV antibody detection probabilities in birds sampled in Atlanta, Georgia and Chicago, Illinois (2010–2012)

Overlap 2010–2012					
Variable	Estimate	SE	*z* Value	Pr(>|*z*|)
Intercept	−6.16 (−8.23, −4.10)	1.06	−5.84	5.4e−9
Year: 2011	0.79 (0.21, 1.40)	0.30	2.66	0.008
Year: 2012	3.15 (2.42, 3.89)	0.37	8.42	<2e−16
Age	−12.9 (−17.5, −8.21)	2.37	−5.42	6.0e−8
Month	0.28 (0.05, 0.50)	0.11	2.4	0.02
Month by age	1.49 (0.90, 2.07)	0.30	5.00	5.8e−7	
Random effects	Name	Variance	SD	
Location: species	Intercept	0.009	0.09	
Species	Intercept	1.58	1.26		

WNV detection was the response term, total monthly trapping effort was included as an intercept offset, month, age, month-by-age interaction, and year (categorical with reference “2010”) were fixed effect terms, and location nested within species was a random effect term. Pr(>|z|) = *P* value.

The predicted probabilities of WNV detection in the five sentinel species in the overlap GLMMs were consistent with the city-specific GLMMs (Supplemental Figure 4). Additionally, species was the greatest source of variance among the random effect terms in the overlap GLMMs (Table 3). Overall, NOCA, COGR, and EUST were the most significant sources of among-species variance (Supplemental Figure 5); also, CIs of the odds ratio estimates did not overlap between NOCA and AMRO, indicating that WNV seroprevalence was statistically different between these two species. Overall, little variance was attributable to the location by species nested random effect term (Table 3), indicating there was little variation in location effects on WNV serostatus between species when comparing serostatus estimates in Atlanta and Chicago from 2010 to 2012.

## DISCUSSION

Our results reveal general trends in WNV seroprevalence common to both Chicago and Atlanta. Overall, species identity was a greater source of variation in WNV seroprevalence in each city than location and year of sampling. Additionally, year-round resident and summer breeding species had higher seroprevalence rates than migrant and winter residents. This result supports prior research on the influence of host community composition on patterns of WNV transmission.^3^^,^^7^^,^^22^ Our long-term results were also consistent with previous short-term studies revealing a greater seroprevalence of WNV in northern cardinals than in all other investigated species, and a lower prevalence of WNV antibodies in urban natural areas than in more densely urbanized residential locations.^4^^,^^15^^,^^17^^,^^37^^–^^39^ Notably, our results highlight possible differences in WNV avian exposure rates between the two cities, including higher overall WNV seroprevalence rates in birds in Atlanta, and differences in the importance of habitat and seasonality for each city. The greater seroprevalence of WNV in Atlanta than in Chicago across all levels of analyses was unexpected. This primary difference in WNV seroprevalence estimates, plus the differences in variation explained by location and year of sampling, could be driven by variation in sampling methodologies, our chosen analytical methods, *Cx. pipiens* complex mosquito population dynamics and blood feeding preferences, and/or differences in the seasonality of WNV in the Southeast and Midwest.

The sampling methodologies in each city varied slightly, and these differences in sample design could have influenced our results. One such difference in each dataset is the level of spatial and temporal replication. The Chicago portion of the dataset is much larger, reflecting an overall greater sampling effort in a greater variety of habitats capturing a greater number of species. However, capture rates per sampling event were similar between cities. Habitat replication in Chicago focused on a variety of sites within urban settings, which could have minimized the influence of habitat on seroprevalence estimates. Additionally, city-specific analyses revealed that location of sampling, which was modeled as a random effect to account for inherent differences among sites, contributed less to variance in WNV seroprevalence in Chicago than year of sampling; rather, location of sampling was a more important source of variance in Atlanta. The importance of location as a source of variance in Atlanta can be mostly explained by site selection: from 2010 to 2012, our field sampling design specifically focused on variability of WNV transmission in urban microhabitats such that site selection focused on completely wooded, residential, and urban green spaces. Despite these sampling differences, the greater prevalence of avian WNV antibodies in Atlanta compared with Chicago was robust across all data restrictions.

Temporal differences between the datasets could then explain some of the difference in antibody prevalence between the cities. Much of the Chicago dataset covers the years closer to the initial establishment of WNV in Chicago (∼2002). Large-scale invasion of the then novel WNV could explain the high prevalence of WNV antibodies in birds in 2005 followed by the sharp drop in prevalence (and especially in hatch year birds’ incidence) in 2006.^40^ However, several regions in the US have revealed large WNV enzootic and epidemics many years following initial establishment, as occurred in 2012.^41^ Similar dynamics following initial WNV invasion into the Southeast could not be assessed for the Atlanta data, the collection of which began a decade later. In contrast to Chicago, there was consistently very little difference in antibody prevalence between years in the Atlanta dataset. This could be due to the generally longer enzootic transmission season in Atlanta,^20^ which reduces the influence of interannual variability in climatic influences on WNV transmission.

It is also important to consider the impact of our data restrictions on our detected patterns. Multiple species that have been shown to be important in each city were excluded from our final analyses. In Chicago, house sparrows and American robins are two species that significantly contribute to the WNV force of infection in mosquito populations^6^ whereas in Atlanta, members of the Mimidae family (including northern mockingbirds and brown thrashers), which are considered only mildly competent hosts of WNV, are possible supersuppressors of WNV epidemic transmission.^4^ In the absence of our data restrictions, the inclusion of all species in our preliminary analyses only heightened the difference in overall WNV seroprevalence between cities. Our comparisons of seroprevalence among species and between groups of species with different residence status consistently demonstrated higher WNV prevalence in Atlanta species compared with Chicago species. Despite excluding certain species in the final analyses, the detected patterns of WNV seroprevalence among the five sentinel species in the city-specific GLMMs were remarkably similar and were consistent with the results from our initial proportions tests.

A key difference between cities is that *Cx. pipiens pipiens* is the primary vector in Chicago, whereas *Cx. quinquefasciatus* is the primary vector in Atlanta; the *Cx. pipiens* form *molestus* also exists in Chicago. All three taxa are considered biotypes in the *Cx. pipiens* complex, and all three are competent WNV vectors. It is widely known that blood feeding behaviors vary widely between these subspecies: *Cx. pipiens pipiens* is considered an ornithophilic feeder whereas *Cx. quinquefasciatus* is considered a more generalist blood feeder.^42^^,^^43^ However, a recent review of vector contributions to WNV transmission determined that avian feeding frequencies are, on average, similar between *Cx. pipiens pipiens* and *Cx. quinquefasciatus*.^44^ Prior research in Chicago has linked a greater prevalence of mammalian feeding in *Cx. pipiens* individuals that show genetic admixture signatures with *molestus*^45^; a similar genetic association with hybridization with *Cx. quinquefasciatus* has not been explored. A theoretical study of WNV transmission by *Cx. pipiens* complex mosquitoes suggests that transmission rates would decrease, and human spillover would increase, with a greater frequency of generalist, mammalian biting.^46^ Thus, one might predict a lower antibody prevalence in birds in Atlanta than in Chicago (or a greater incidence of WNV in humans) based on general patterns of host choice among members of the *Cx. pipiens* complex, but this was not the pattern observed by our study. Although other vector species may contribute to WNV in each city, such as *Cx. restuans* and *Aedes albopictus*, there is little evidence to support these species as contributing to WNV during periods of enzootic or epidemic transmission.^25^^,^^47^^,^^48^

The pattern of more consistent enzootic activity in the southern US and less consistent but larger epizootics in the northern US has been observed for other zoonoses, such as epizootic hemorrhagic disease virus (EHDV), which is vectored by *Culicoides* biting midges among mammals, primarily deer.^49^^,^^50^ Similar to EHDV regional dynamics, our avian WNV seroprevalence data appear to display less intense amplification events in subtropical and tropical regions of the Americas where WNV has been introduced, and spillover to humans rarely occurs.^51^^–^^53^ This suggests that latitudinal gradients of WNV seasonality driven by the *Cx. pipiens* complex may be related to observed differences in Chicago and Atlanta WNV transmission cycles. Differences in the length of time that temperatures are suitable for WNV replication and transmission by mosquitoes^20^^,^^27^^,^^54^^–^^56^ may influence WNV transmission efficiency among birds in each city, with greater overall opportunities for WNV transmission in Atlanta. Differences in WNV seroprevalence may also be due to differences in avian breeding seasons between each city. The bird breeding season at a higher latitude in Chicago for the primary enzootic hosts of WNV would be temporally restricted compared with the lower latitude of Atlanta. This constrained bird breeding season in Chicago could result in a larger pulse of susceptible juvenile birds, which has been shown to fuel the WNV amplification cycle in Chicago.^15^ Indeed, our predicted juvenile bird seroconversion starts earlier in Atlanta than in Chicago, and the rate of increase is higher in Chicago compared with Atlanta.

While overall WNV seroprevalence was higher in Atlanta than Chicago, spillover of WNV to humans in the Southeastern US is much less common and more sporadic than in the Upper Midwest. Between 2010 and 2016 in Fulton County, Georgia (where Atlanta is located; population ∼1.0 million), a total of only 19 human cases (clinical and neuroinvasive) were reported, with neuroinvasive disease rates varying between 0.0 and 0.6 per 100,000 residents during this time period. In contrast, between 2005 and 2012 in Cook County, Illinois (where Chicago is located; population ∼5.2 million), 588 total human cases were reported, with neuroinvasive disease case rates varying between 0.41 and 2.31 per 100,000 residents. Other metrics associated with WNV transmission such as drought and high summer temperatures, human behavior, and WNV infection rates in mosquitoes have been linked to human spillover,^57^^–^^59^ and they are likely more relevant to predicting WNV spillover into humans than avian serological data.

## Supplemental files


Supplemental materials
