# Supplementary material for: Multi-Year Comparison of Community- and Species-Level West Nile Virus Antibody Prevalence in Birds from Atlanta, Georgia and Chicago, Illinois, 2005–2016
Source: Am J Trop Med Hyg. 2022 Dec 26;108(2):366–76. doi: 10.4269/ajtmh.21-1086 (PMC9896344; doi:10.4269/ajtmh.21-1086)
Supplement: Supplementary file 1 [file tpmd211086.SD1.pdf]

Supporting Information for:

Title: Multi-year comparison of community- and species-level West Nile virus antibody prevalence in birds from Atlanta, Georgia and Chicago, Illinois USA 2005 - 2016

Authors: Joseph R. McMillan[1], Gabriel L. Hamer[2], Rebecca Levine[1], Daniel G. Mead[3], Lance A. Waller[1,4], Tony Goldberg[5], Edward Walker[6], Jeffrey Brawn[7], Marilyn O. Ruiz[8], Uriel Kitron[1,9], Gonzalo Vazquez-Prokopec[1,9**]

Author Affiliations:
[1]Program in Population Biology, Ecology and Evolution, Emory University, Atlanta, GA, USA
[2]Department of Entomology, Texas A&M University, College Station, TX, USA
[3]Southeastern Cooperative Wildlife Disease Study, University of Georgia, Athens, GA, USA
[4]Department of Biostatistics and Bioinformatics, Rollins School of Public Health, Emory University
[5]Department of Pathobiological Sciences, School of Veterinary Medicine, University of Wisconsin-Madison, Madison, WI, USA
[6]Department of Microbiology and Molecular Genetics, Michigan State University, East Lansing, MI, USA
[7]Department of Natural Resources and Environmental Sciences, University of Illinois Champaign-Urbana, Urbana, IL, USA
[8]*Deceased* – Department of Pathobiology, University of Illinois Champaign-Urbana
[9]Department of Environmental Sciences, Emory University

Author Email: jrmcmil@emory.edu, ghamer@tamu.edu, rclevin@alum.emory.edu, dmead@uga.edu, lwaller@emory.edu, tony.goldberg@wisc.edu, walker@msu.edu, jbrawn@illinois.edu, mailto:ukitron@emory.edu, gmvazqu@emory.edu

*****Corresponding Author**
Gonzalo Vazquez-Prokopec
400 Dowman Drive
Math and Science Center, 5th floor, Suite E530
Department of Environmental Sciences, Emory University.
Atlanta, GA, 30322, USA.
Tel: (404) 727-4217; fax: (404) 727-4448.
Email: gmvazqu@emory.edu

Keywords: antibody, West Nile virus, serology, northern cardinal, American robin

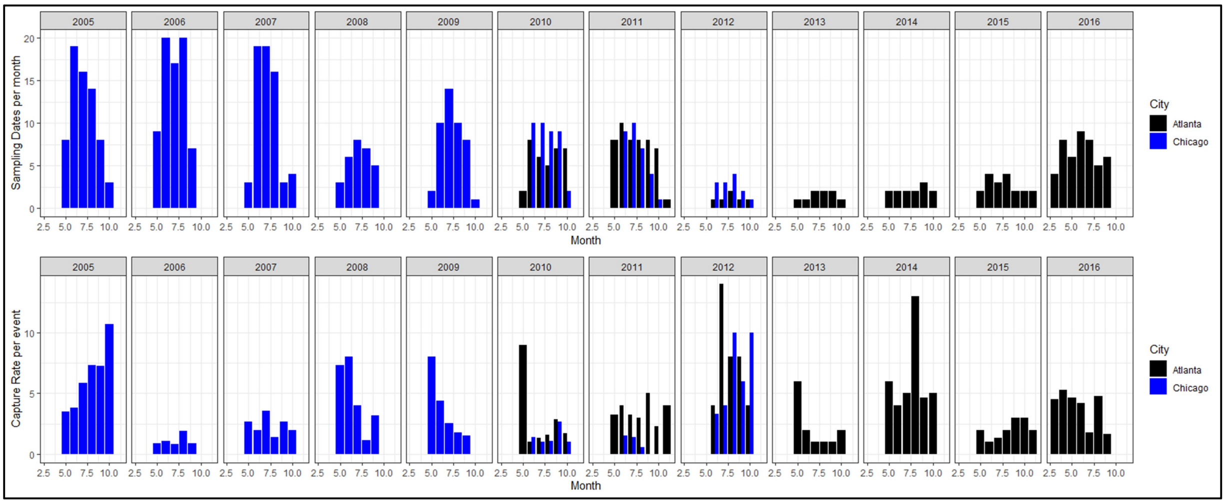

S. Figure 1. Sampling effort and capture rates per sampling event in Atlanta, GA and Chicago, IL 2010 - 2012.

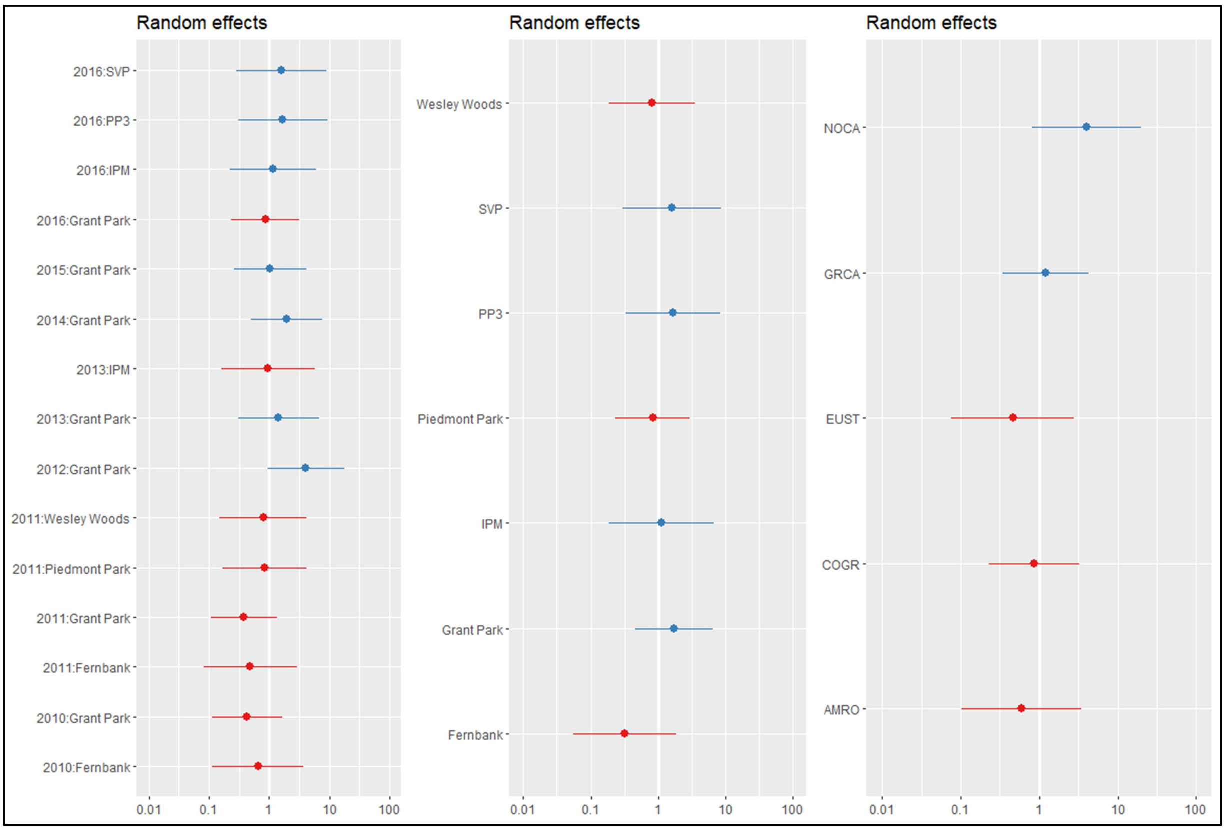

S. Figure 2. The random effect predictions – shown as an odds ratio - of a bird testing positive for West Nile virus antibodies in Atlanta, GA 2010 - 2016. Predictions were generated from a binomial-error generalized linear mixed effect model (GLMM) with West Nile virus antibody detection as the response term, Month, Age, and a Month by Age interaction term as fixed effects, Species as a crossed random effect, and Year and Location as nested random effects. The left plot displays the odds for the nested effect of Years by Site; the middle plot displays the odds for the crossed effect of Site; the right plot displays the odds for the crossed effect of Species. Colors indicate whether the estimate is below (red) or above (blue) 1. Points represent the estimate while lines represent the 95% CI of the estimate. 95% CI that do not include unity would be considered significant. 95%CI that do not overlap between random effect terms would also be considered significant.

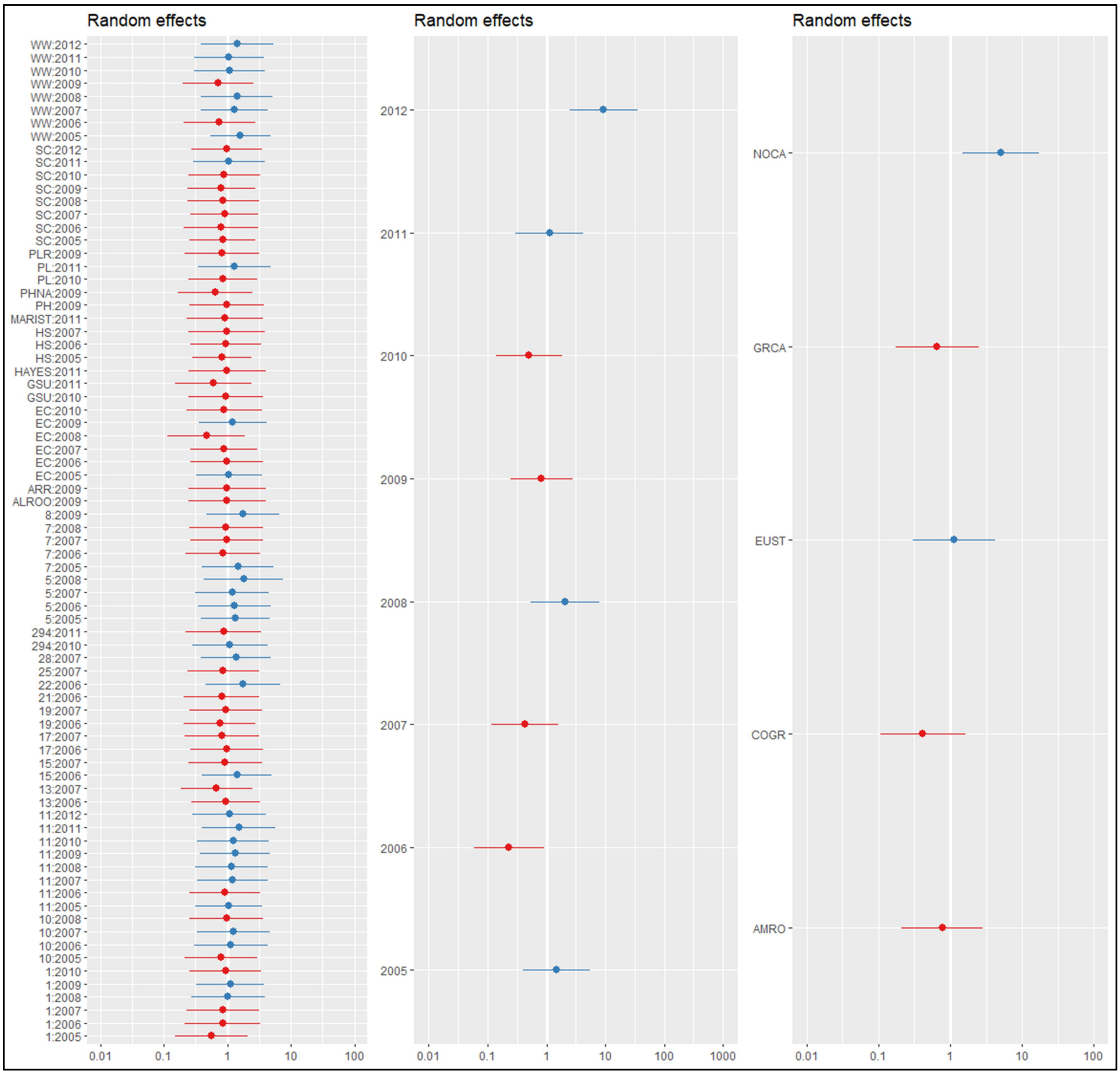

S. Figure 3. The random effect predictions – shown as an odds ratio - of a bird testing positive for West Nile virus antibodies in Chicago, IL 2005 - 2012. Predictions were generated from a binomial-error generalized linear mixed effect model (GLMM) with West Nile virus antibody detection as the response term, Month, Age, and a Month by Age interaction term as fixed effects, Species as a crossed random effect, and Location and Year as nested random effects. The left plot displays the odds for the nested effect of Site by Year; the middle plot displays the odds for the crossed effect of Year; the right plot displays the odds for the crossed effect of Species. Colors indicate whether the estimate is below (red) or above (blue) 1. Points represent the estimate while lines represent the 95% CI of the estimate. 95% CI that do not include unity would be considered significant. 95%CI that do not overlap between random effect terms would also be considered significant.

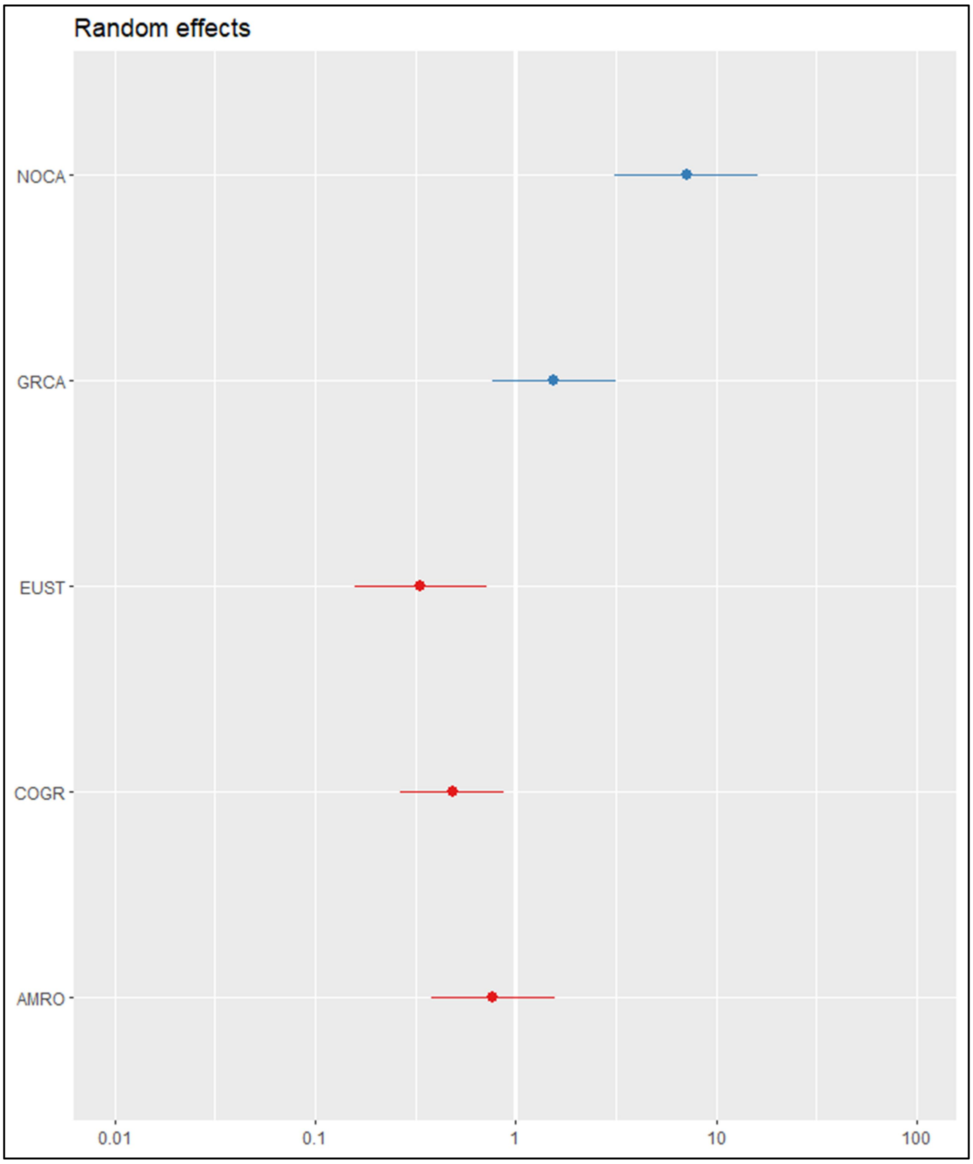

S. Figure 4. The random effect predictions – shown as an odds ratio - of a bird testing positive for West Nile virus antibodies in Atlanta, GA and Chicago, IL 2010 - 2012. Predictions were generated from a binomial-error generalized linear mixed effect model (GLMM) with West Nile virus antibody detection as the response term, Month, Age, Year, and a Month by Age interaction term as fixed effects, Species as a crossed random effect, and Location and Species as nested random effects. The plot displays the odds for the crossed effect of Species. Colors indicate whether the estimate is below (red) or above (blue) 1. Points represent the estimate while lines represent the 95% CI of the estimate. 95% CI that do not include unity would be considered significant. 95%CI that do not overlap between random effect terms would also be considered significant.

Supporting Information for:

Title: Multi-year comparison of community- and species-level West Nile virus antibody prevalence in birds from Atlanta, Georgia and Chicago, Illinois USA 2005 - 2016

Authors: Joseph R. McMillan[1], Gabriel L. Hamer[2], Rebecca Levine[1], Daniel G. Mead[3], Lance A. Waller[1,4], Tony Goldberg[5], Edward Walker[6], Jeffrey Brawn[7], Marilyn O. Ruiz[8], Uriel Kitron[1,9], Gonzalo Vazquez-Prokopec[1,9**]

Author Affiliations:
[1]Program in Population Biology, Ecology and Evolution, Emory University, Atlanta, GA, USA
[2]Department of Entomology, Texas A&M University, College Station, TX, USA
[3]Southeastern Cooperative Wildlife Disease Study, University of Georgia, Athens, GA, USA
[4]Department of Biostatistics and Bioinformatics, Rollins School of Public Health, Emory University
[5]Department of Pathobiological Sciences, School of Veterinary Medicine, University of Wisconsin-Madison, Madison, WI, USA
[6]Department of Microbiology and Molecular Genetics, Michigan State University, East Lansing, MI, USA
[7]Department of Natural Resources and Environmental Sciences, University of Illinois Champaign-Urbana, Urbana, IL, USA
[8]*Deceased* – Department of Pathobiology, University of Illinois Champaign-Urbana
[9]Department of Environmental Sciences, Emory University

Author Email: jrmcmil@emory.edu, ghamer@tamu.edu, rclevin@alum.emory.edu, dmead@uga.edu, lwaller@emory.edu, tony.goldberg@wisc.edu, walker@msu.edu, jbrawn@illinois.edu, mailto:ukitron@emory.edu, gmvazqu@emory.edu

*****Corresponding Author**
Gonzalo Vazquez-Prokopec
400 Dowman Drive
Math and Science Center, 5th floor, Suite E530
Department of Environmental Sciences, Emory University.
Atlanta, GA, 30322, USA.
Tel: (404) 727-4217; fax: (404) 727-4448.
Email: gmvazqu@emory.edu

Keywords: antibody, West Nile virus, serology, northern cardinal, American robin

Species	Atlanta, GA 2010 - 2016								Chicago, IL 2005 - 2012							
	Status	Sample Size	Locations	Years	Adults	WNV seroprevalence	Hatch years	WNV seroincidence	Status	Sample Size	Locations	Years	Adults	WNV seroprevalence	Hatch years	WNV seroincidence
AMGO									Resident	258	17	8	240	6%	18	0%
AMRE									Summer	6	5	4	5	0%	1	0%
AMRO	Resident	237	11	7	145	28%	92	12%	Resident	1005	28	8	473	14%	532	4%
AMWO									Summer	1	1	1	0		1	0%
ATSP									Winter	1	1	1	1	0%		
BAOR									Summer	31	7	7	26	8%	5	20%
BARS									Summer	6	1	2	2	0%	4	0%
BAWW	Summer	1	1	1	1	0%			Summer	3	3	3	1	0%	2	0%
BBCU									Summer	2	1	2			2	0%
BCCH									Resident	26	9	7	21	0%	5	0%
BHCO	Resident	5	2	3	5	20%			Resident	56	10	8	43	5%	13	15%
BLJA	Resident	22	7	6	15	60%	7	29%	Resident	22	10	5	11	36%	11	9%
BLPW									Migrant	2	1	2	1	0%	1	0%
BRTH	Resident	71	9	7	58	48%	13	0%	Summer	6	3	4	5	40%	1	0%
BTBW									Migrant	2	1	1	2	0%		
CARW	Resident	58	12	6	48	23%	10	10%	Resident	2	2	2	2	0%		
CAWA									Migrant	1	1	1	1	0%		
CEDW	Winter	1	1	1	1	0%			Resident	17	6	5	16	7%	1	0%
CHSP									Summer	21	9	6	17	12%	4	0%
COGR	Resident	39	5	7	33	39%	6	0%	Resident	92	13	8	71	7%	21	0%
COYE									Summer	7	3	4	6	17%	1	0%
CSWA	Migrant	1	1	1			1	0%								
DOWO	Resident	4	3	3	3	33%	1	0%	Resident	43	14	8	29	7%	14	7%
EABL	Resident	15	4	4	5	20%	10	10%								
EAPH	Resident	12	4	6	7	0%	5	0%	Summer	1	1	1	1	0%		
EATO	Resident	20	7	6	12	8%	8	0%	Summer	2	1	1	2	0%		
EAWP	Summer	1	1	1	1	0%			Summer	1	1	1			1	0%
EUST	Resident	40	5	6	19	0%	21	5%	Resident	122	14	8	47	11%	75	5%
FOSP									Migrant	3	1	2			3	0%
GCFL	Summer	3	3	2	3	0%			Summer	3	3	2	3	0%		
GCTH	Migrant	7	4	4	6	0%	1	0%	Migrant	18	7	8	9	11%	9	11%

GRCA	Summer	90	8	7	66	41%	24	25%	Summer	375	21	8	232	11%	143	13%
HAWO									Resident	1	1	1			1	0%
HETH									Migrant	8	3	3	2	0%	6	0%
HOFI	Resident	5	4	3	4	25%	1	0%	Resident	135	12	7	52	23%	83	10%
HOSP	Resident	2	2	2	2	50%			Resident	1882	22	8	778	12%	1104	7%
HOWA	Summer	5	4	1	3	0%	2	0%								
HOWR	Resident	1	1	1	1	100%			Summer	9	5	6	5	0%	4	0%
INBU	Summer	1	1	1			1	0%	Summer	16	9	7	14	0%	2	0%
KEWA	Summer	1	1	1	1	0%										
KILL									Summer	1	1	1	1	0%		
LEFL									Summer	1	1	1	1	0%		
LISP									Migrant	3	3	3	2	0%	1	0%
LOWA									Summer	1	1	1	1	0%		
MALL									Resident	4	1	1	4	0%		
MAWA	Migrant	1	1	1	1	0%			Migrant	7	3	3	3	0%	4	0%
MODO	Resident	1	1	1			1	0%	Resident	45	10	5	23	52%	22	27%
MOWA									Migrant	1	1	1	1	0%		
MYWA									Migrant	17	5	3	7	0%	10	0%
NAWA									Migrant	2	2	2	1	0%	1	0%
NOCA	Resident	183	12	7	121	61%	62	32%	Resident	244	22	8	137	57%	107	36%
NOMO	Resident	93	7	7	51	55%	42	40%								
NOWA	Migrant	6	3	3	6	0%			Migrant	35	8	7	23	0%	12	0%
NRWS									Summer	1	1	1	1	0%		
OROR									Summer	3	1	1	3	0%		
OSFL									Migrant	1	1	1	1	0%		
OVEN	Summer	2	2	1	2	0%			Summer	31	11	7	13	0%	18	0%
RBGR	Migrant	1	1	1			1	0%	Summer	3	3	2			3	0%
RBWO	Resident	2	2	2	1	0%	1	0%								
REVI	Summer	4	1	2	4	0%			Summer	12	7	6	12	0%		
RHWO	Resident	1	1	1	1	0%										
RWBL	Resident	1	1	1	1	0%			Resident	142	6	8	127	9%	15	13%
SAVS									Summer	2	1	2			2	0%
SCJU									Winter	9	5	3	4	0%	5	0%
SCTA									Summer	1	1	1			1	0%

SOSP	Resident	19	4	5	12	0%	7	0%	Resident	203	11	8	116	3%	87	1%
SWSP									Winter	1	1	1			1	0%
SWTH	Migrant	23	6	6	20	5%	3	0%	Migrant	116	11	8	45	0%	71	1%
TRES									Summer	6	2	2	5	0%	1	0%
TUTI	Resident	11	3	2	9	0%	2	0%								
VEER	Migrant	1	1	1	1	0%			Summer	6	5	3	4	0%	2	0%
WAVI									Summer	27	4	7	19	0%	8	0%
WBNU	Resident	9	4	4	9	22%			Resident	1	1	1	1	0%		
WCSP									Winter	9	4	4	5	20%	4	0%
WIFL									Summer	31	4	4	29	0%	2	50%
WOTH	Summer	7	3	2	6	0%	1	0%	Summer	3	2	2	3	0%		
WPWA									Migrant	3	1	1			3	0%
WTSP	Winter	11	4	3	10	0%	1	0%	Winter	61	10	6	28	4%	33	0%
YBCH	Summer	1	1	1	1	100%										
YBFL									Migrant	3	2	1	3	0%		
YSFL	Resident	3	2	3	3	0%			Resident	10	6	4	8	0%	2	0%
Totals and Averages		1022	3.66	3.2	698	18%	324	6%		5232	5.36	3.71	2749	6%	2483	4%

S. Table 1. Collection and West Nile virus seroprevalence summaries for all bird species captured in Atlanta, Georgia (2010 – 2016) and Chicago, Illinois (2005 – 2012). The Species column contains the 4-letter alpha codes used by US-based bird banders to record and submit banding information. Actual species and common names can be found by performing an Internet search with the alpha codes or referencing an alpha-code manual.
